# The Genetics of Reading Disabilities: From Phenotypes to Candidate Genes

**DOI:** 10.3389/fpsyg.2012.00601

**Published:** 2013-01-07

**Authors:** Wendy H. Raskind, Beate Peter, Todd Richards, Mark M. Eckert, Virginia W. Berninger

**Affiliations:** ^1^Department of Medicine, University of WashingtonSeattle, WA, USA; ^2^Department of Psychiatry and Behavioral Sciences, University of WashingtonSeattle, WA, USA; ^3^Department of Radiology, University of WashingtonSeattle, WA, USA; ^4^Department of Speech and Hearing Sciences, University of WashingtonSeattle, WA, USA; ^5^Department of Otolaryngology/Head and Neck Surgery, Medical University of South CarolinaCharleston, SC, USA; ^6^Department of Health Science and Research, Medical University of South CarolinaCharleston, SC, USA; ^7^Department of Educational Psychology, University of WashingtonSeattle, WA, USA

**Keywords:** dyslexia, attention deficit/hyperactivity disorder, language impairment, speech sound disorder, genetics, candidate genes, reading impairment, brain imaging

## Abstract

This article provides an overview of (a) issues in definition and diagnosis of specific reading disabilities at the behavioral level that may occur in different constellations of developmental and phenotypic profiles (patterns); (b) rapidly expanding research on genetic heterogeneity and gene candidates for dyslexia and other reading disabilities; (c) emerging research on gene-brain relationships; and (d) current understanding of epigenetic mechanisms whereby environmental events may alter behavioral expression of genetic variations. A glossary of genetic terms (denoted by bold font) is provided for readers not familiar with the technical terms.

## Complex Phenotype of Specific Reading Disabilities

Specific reading disabilities are genetically and phenotypically **complex** neurobehavioral disorders that affect 5–10% of school-aged children, depending on criteria used to make the diagnosis (Shaywitz et al., 1990b; Katusic et al., 2001). Although anyone with a developmental disability is likely to have difficulty learning to read, specific reading disability is reserved for those whose overall developmental falls at least within the lower limits of the normal range and thus the reading problem cannot be attributed to the developmental disability. Some specific reading disabilities are associated with difficulty decoding unfamiliar words and/or difficulty in recognizing real words encountered before. To assess decoding, pseudowords or non-words that can be pronounced by applying alphabetic principle but are not associated with a conventional meaning are used, whereas to assess real word reading, written words that are associated with one or more conventional meanings are used.

More research has been conducted with dyslexia than other kinds of specific reading disabilities. According to the International Dyslexia Association (Lyon et al., 2003) dyslexia is characterized by a struggle in acquiring written language at the word level, showing deficits in accurate and/or fluent word recognition, decoding, and spelling. Secondary effects on comprehension and reduced reading experience may result, which, in turn, can lead to impoverished vocabulary and the general knowledge base.

A phonological processing deficit, interpreted as evidence of disordered internal representation of speech sounds, is often associated with dyslexia. For example, dissecting words into individual sounds and making changes to their sequence is difficult for individuals with dyslexia, compared to individuals without dyslexia (Morais et al., 1986; Fletcher et al., 1994; Anthony et al., 2002). Phonological short-term memory may be impaired as well, which is assessed with non-word imitation tasks, where non-words such as “woodoip” or “bamadana” are presented as targets to be repeated (Wagner et al., 1999). Naming (producing spoken names) for visual stimuli (e.g., colors, objects, letters, numerals) rapidly (Rapid Automatic Naming, RAN) is also frequently impaired in individuals with dyslexia (Wolf and Bowers, 1999). For instance, children with dyslexia were slower and less accurate than children without dyslexia in RAN for pictures (Denckla and Rudel, 1976; Catts, 1986; Wolf and Bowers, 1999). Whereas in the past, deficits in phonological processing ability were thought to be caused by deficits in auditory perception, especially when rapid acoustic transitions were involved (Tallal, 1980; Farmer and Klein, 1993), more recent research shows that extraction of linguistic units larger than phonemes, e.g., syllables and words, from the speech stream may also be deficient in individuals with specific reading disabilities (Johnson et al., 2011) who may process other acoustic features such as cues for voice identification less efficiently (Perrachione et al., 2011).

Processes beyond those involving speech sounds appear to be implicated. Evidence has accumulated that working memory (Swanson and Berninger, 1995; Swanson and Siegel, 2001) and central executive functions (Lyon and Kranegor, 1996; Swanson, 2000; Berninger et al., 2006) in working memory are impaired in reading disabilities in general and dyslexia in particular. Children with oral language impairment (LI; Miller et al., 2001; Leonard et al., 2007) and written LIs including dyslexia (Catts et al., 2002b; Smith et al., 2008; Peter et al., 2011a) may also have slowed processing speeds. Many studies have found an excess of males, with male:female ratios typically ranging from 1:5 to 3:1 in reading disability (Flannery et al., 2000; Katusic et al., 2001; Rutter et al., 2004), but more recently several studies have shown that the gender difference may be specific to the impaired spelling and related writing skills in dyslexia, not the reading skills (for review, see Berninger et al., 2008). Ascertainment bias may account for a portion of this sex differential (Shaywitz et al., 1990a). With appropriate educational intervention, most affected individuals eventually achieve some proficiency in reading and writing skills, but deficits in phonological decoding, fluent oral reading, and spelling often persist into adulthood even in those whose word reading problems appear to be “compensated” (Bruck, 1990, 1992, 1993; Pennington et al., 1990; Wilson and Lesaux, 2001; Berninger et al., 2006).

Several factors complicate studies of the etiology of reading disabilities in general and dyslexia in particular. First, problems with reading are not limited to dyslexia. Other developmental disabilities, such as LI and speech sound disorder (SSD), share reading impairment (Catts et al., 2002a, 2008; Peterson et al., 2009; Landerl and Moll, 2010), and study populations may contain mixtures of individuals with different underlying disorders. Second, reading disability may co-occur with these other disorders or with attention deficit disorder, all of which also have a genetic basis (Pennington and Bishop, 2009), and such comorbidity may confound the parsing of etiologies. Third, because the distributions of reading ability relative to age or IQ are continuous, the setting of a discrete threshold for dyslexia is somewhat arbitrary and varies among different research groups.

One emerging approach to dealing with this lack of homogeneity regarding kinds of reading disabilities is to differentiate between developmental disabilities, specific learning disabilities, and **endophenotypes**. For example, in a special issue devoted to improving communication and collaboration among speech and language specialists, psychologists, and educators, a model was proposed for defining and diagnosing disabilities based on profiles (patterns of variables or constellations) rather than a single variable out of context of other related, relevant variables (Silliman and Berninger, 2011). Evidence exists for five domains of development (each related to different brain systems): (a) cognition and memory; (b) receptive and expressive language; (c) sensory and motor systems; (d) social and emotional systems; and (e) attention and executive function systems. Individuals who fall outside the normal range in one or more but not all developmental domains have specific developmental disabilities (SDDs) and those who fall outside the normal range in all developmental domains have pervasive developmental disabilities (PDDs). Sometimes diagnosed PDDs or SDDs are associated with specific neurogenetic disorders with characteristic phenotypes, for example, fragile X or Williams syndrome (Batshaw et al., 2007). For students without SDDs or PDDs, behavioral profiles are assessed for specific aural language skills (language by ear), specific oral language skills (language by mouth), specific reading skills (language by eye), and specific writing skills (language by hand; Liberman, 1999; Silliman and Berninger, 2011), each of which has different levels of language (subword, word, and text) that should be differentiated from speech sound processing and production/articulation disorders (Berninger and Niedo, 2012). Evidence is accumulating that three kinds of specific written language disabilities – dysgraphia (impaired handwriting), dyslexia (impaired word decoding and spelling), and selective language disorder (oral and written language learning disability, OWL LD) can be identified and differentiated on the basis of which working memory component/s is/are impaired (spoken and written word form and syntactic storage and processing units; phonological and orthographic loops; and supervisory attention/executive functions like selective attention, switching attention, and sustained attention). Each working memory component could be associated with different genetic etiologies (Berninger and Richards, 2010).

Given this potential confounding of impaired reading found in many kinds of developmental and learning disabilities, it is often difficult to determine whether samples across different genetic studies include the same kinds of reading disabilities or patterns of impairments in individuals with reading disabilities. Nevertheless, the evidence to date on impaired reading (word decoding, word recognition, and reading comprehension) and writing is yielding new knowledge about the biological basis of developmental and learning disabilities. As progress is also made toward a closer description of the various observable traits at the behavioral level, the relationships between various candidate genes, whether acting alone or together, and observable forms of reading disorders may become clearer.

## Genetic Influences on Specific Learning Disabilities

### Genetic basis of reading disabilities

In the context of this overview, it is important to keep in mind that reading disability and dyslexia are often used interchangeably and samples may include reading disabilities related to a variety of language and speech impairments. Genetic influences on reading ability have been demonstrated (Gilger et al., 1994; Reynolds et al., 1996; Harlaar et al., 2005; Davis et al., 2009; Lind et al., 2010) and multiple lines of evidence have led to the consensus that reading impairment has a genetic basis. The earliest observations of **familial clustering** and increased **recurrence risk** of dyslexia in relatives date from the early 1900s (Hinshelwood, 1907; Stephenson, 1907; Hallgren, 1950; DeFries et al., 1978). Twin and adoption studies showed that the familial clustering reflects shared genetic factors more than shared environment (DeFries et al., 1987; Stevenson et al., 1987; Pennington et al., 1991; Gayán and Olson, 2001, 2003; Wadsworth et al., 2002). Most **heritability** estimates range from 40 to 60%. Dyslexia and some component **phenotypes** aggregate in families in a manner consistent with a genetic etiology (Raskind et al., 2000; Hsu et al., 2002) and allow **models of transmission** to be fit (Pennington et al., 1991; Wijsman et al., 2000; Chapman et al., 2003).

### Genetic heterogeneity

Although the heritability of a specific reading disability such as dyslexia has been shown to be high, it is clear that dyslexia is a genetically **heterogeneous** disorder, and for most individuals it is highly likely that more than one genetic factor interact to cause the susceptibility. By both **targeted** and **genome-wide** studies, more than 20 genes and locations have been associated with dyslexia, including nine that have been designated dyslexia loci DYX1–9 by the Human Gene Nomenclature Committee www.genenames.org/. Similar studies have identified candidate locations for genes that modulate LI and other related disabilities (Barry et al., 2007; Conti-Ramsden et al., 2007; Hayiou-Thomas, 2008; Newbury and Monaco, 2010; Newbury et al., 2010; Willcutt et al., 2010). Furthermore, there is evidence for overlapping genetic risk factors among speech disorders, LI, and reading disability (Caylak, 2007; Pennington and Bishop, 2009; Newbury et al., 2011), which may be best understood in the context of shared risks and unique contributors that lead to the specific behavioral profile. We provide a synopsis of these putative genetic loci.

To date, eleven genome-wide scans for dyslexia (Fagerheim et al., 1999; Nopola-Hemmi et al., 2001; Fisher et al., 2002a; Kaminen et al., 2003; de Kovel et al., 2004; Raskind et al., 2005; Igo et al., 2006; Brkanac et al., 2008; König et al., 2011; Rubenstein et al., 2011; Svensson et al., 2011) and genome-wide association scans for early reading disability (Meaburn et al., 2008) and an electrophysiologic measure related to speech processing (Roeske et al., 2011) have been reported. Not all studies have obtained significant results (Meaburn et al., 2008; Svensson et al., 2011).

*The DYX1 locus on chromosome 15* (**MIM** 127700) was first proposed by Smith et al. (1983) in a study of nine multigenerational families in which a **centromeric cytogenetic heteromorphism** appeared linked to dyslexia defined categorically. When the study was extended the **logarithm of odds (LOD)** score decreased (Fain et al., 1985; Lubs et al., 1991) and an independent study failed to detect **linkage** to this location even though it included a large family that had provided almost all the original chromosome 15 signal (Rabin et al., 1993). However, by targeted analyses of chromosome 15q as well as genome-wide approaches, multiple groups obtained evidence for a locus more **distal** on the **long arm** (q21) for single word reading and spelling (Grigorenko et al., 1997, 2007; Schulte-Korne et al., 1998; Nöthen et al., 1999; Morris et al., 2000; Chapman et al., 2004; Marino et al., 2004; Bates et al., 2007; Schumacher et al., 2008; Rubenstein et al., 2011).

*Evidence for a locus on chromosome 6p (DYX2)* was originally reported by Cardon et al. (1994, 1995). They targeted the **HLA locus** based on a hypothesis about a relationship between autoimmunity and dyslexia. Since that time, the DYX2 locus on 6p21 (OMIM 600202) has been intensively studied using multiple phenotypes, both categorically defined or modeled as continuous variables, and linkage has been replicated by multiple groups (Grigorenko et al., 1997, 2007; Fisher et al., 1999; Gayan et al., 1999; Petryshen et al., 2000; Kaplan et al., 2002; Marlow et al., 2003; Turic et al., 2003; Deffenbacher et al., 2004; Cope et al., 2005a; Schumacher et al., 2006a). As observed for DYX1, linkage, and **association** studies between dyslexia and DYX2 have not provided uniformly supportive results (Field and Kaplan, 1998; Petryshen et al., 2000; de Kovel et al., 2008). Recently support for an additional dyslexia locus near DYX2 that contributes to a rapid naming phenotype was obtained in a German population (König et al., 2011).

*DYX3 on chromosome 2p15–p16* (OMIM 604254) was first identified in a genome-wide scan in a single large Norwegian family (Fagerheim et al., 1999). Evidence supporting this locus has been reported from studies in the United Kingdom (Fisher et al., 2002a), Canada (Petryshen et al., 2002), Finland (Kaminen et al., 2003), and the Netherlands (de Kovel et al., 2008) but the genetic regions did not consistently overlap.

*The DYX4 locus on chromosome 6q13–q16* has only been reported by one group (Petryshen et al., 2001). In this study of 96 Canadian families, the **parametric** and **non-parametric** LOD scores did not reach significance levels usually set as thresholds and this locus is not listed in the OMIM database.

*The DYX5 locus* (OMIM 606896) was identified in a genome-wide scan in a large Finnish family with impairments in rapid naming, phonological awareness, and verbal short-term memory. Linkage of a categorical dyslexia assessment was detected to the pericentromeric region of chromosome 3p12–q13 (Nopola-Hemmi et al., 2001). In this family, the most severely affected members had poor reading comprehension, a trait that characterizes LI rather than dyslexia (Nopola-Hemmi et al., 2002). This locus has also been implicated in SSD (Stein et al., 2004).

*The DYX6 locus on chromosome 18p11.2* (OMIM 606616) was identified by genome scans for multiple quantitative endophenotypes of dyslexia in independent sib pair cohorts from the United States and the United Kingdom (Fisher et al., 2002a). This study detected many secondary signals that provide support for some of the other loci identified in other studies. At least one other large study was unable to corroborate the chromosome 18 locus (Schumacher et al., 2006b). Interestingly, a study of reading ability in a population not selected with respect to this behavior also detected evidence of linkage to this region (Seshadri et al., 2007).

*The DYX7 locus on chromosome 11p15* was found by a targeted study of the dopamine receptor D4 (DRD4) as a candidate for dyslexia based on its postulated involvement in Attention deficit/hyperactivity disorder (ADHD) and the frequent co-occurrence of the two disorders (Hsiung et al., 2004). This study utilized a slightly larger set of Canadian families that had been used in the linkage study that yielded DYX4. Neither a significant association with DRD4, nor an excess of the DRD4 seven-repeat **allele** associated with ADHD was found in dyslexic subjects, and no other group has reported supportive evidence for this locus, which has not been assigned a locus designation in OMIM.

Using Rh blood type alleles as markers, one research team found suggestive evidence for linkage of dyslexia defined categorically to *chromosome 1p34–p36* (Rabin et al., 1993). A study of Dutch sib pairs, also using a **categorical affectation status**, obtained supportive evidence for this locus, *now designated DYX8* (OMIM 608995; de Kovel et al., 2008).

The final “named” locus, *DYX9*, *on Xq27* was identified through a genome wide scan for a categorical diagnosis of dyslexia in a single Dutch family (de Kovel et al., 2004). A signal was also seen near to this location in the genome scan that identified the 18q locus (Fisher et al., 2002a).

*Other loci that do not have a DYX appellation* have been identified by a variety of approaches, but many have not been corroborated in other subject samples. Utilizing a large set of well-characterized families with dyslexia and modeling the endophenotypes as quantitative traits, we have identified loci that may contribute to pseudoword reading ability on chromosome 2q22.3 (Raskind et al., 2005), real word reading ability on chromosome 13q12 (Igo et al., 2006), phonological memory on chromosomes 4p12 and 12p12–p13 (Brkanac et al., 2008) and spelling performance on chromosomes 2q11–q22, 9q33–q34155, and 15q12–q14 (Rubenstein et al., 2011). By **quantitative transmission disequilibrium testing (QTDT)** and **linear association modeling**, we found evidence that two functionally related genes, *FOXP2* and *CNTNAP2*, that play a role in speech and language impairments, are associated with phonological memory, real word reading rate, and measures of sequential motor ability in our ascertained for dyslexia (Peter et al., 2011b).

In a **genome-wide association study (GWAS)** for mismatch negativity, an electrophysiologic measure related to speech processing, a marker on chromosome 4q32.1 provided significant results in discovery and replication samples. This region does not contain any protein coding genes, but the markers were associated with mRNA expression levels of *SLC2A3* on chromosome 12 that codes for the predominant neuronal glucose transporter (Roeske et al., 2011). The authors speculate that glucose deficits in neurons might cause the attenuated mismatch negativity during passive listening tasks.

For a complex and heterogeneous disorder, it is not surprising that different research groups have identified unique locations and have not always found supportive evidence for locations reported by others. The studies are usually not directly comparable as there are differences in phenotypes evaluated, ascertainment schemes, eligibility criteria, and analysis methods.

## Other Developmental Disorders with Features that Overlap with Dyslexia

### Attention deficit/hyperactivity disorder

Attention deficit/hyperactivity disorder comprises three behaviorally defined subtypes, predominantly inattentive type (ADHD-PI), predominantly hyperactive-impulsive type (ADHD-PHI), and combined type (ADHD-C; Wolraich et al., 2011; Willcutt et al., 2012), although these may not be fixed and stable distinctions for an individual over time (Frick and Nigg, 2012). ADHD is more frequently observed in males than females (Kronenberger and Dunn, 2003). Many individuals with ADHD also exhibit reading impairment, but slowed processing speed characterizes both ADHD and dyslexia, whereas working memory deficits are associated with dyslexia but not ADHD (McGrath et al., 2011). Given the frequent comorbidity, one hypothesis is that there are shared etiologies. Studies on twins support a predominantly genetic basis for the comorbidity, especially for the inattentive subtype of ADHD (Stevenson et al., 1993; Willcutt et al., 2000, 2007; Nigg et al., 2010). Genome-wide linkage scans have identified multiple loci that may harbor genes for ADHD (Fisher et al., 2002b; Bakker et al., 2003; Ogdie et al., 2003; Arcos-Burgos et al., 2004; Asherson et al., 2008; Faraone et al., 2008; Zhou et al., 2008; Frazier-Wood et al., 2012). Review of the research to date shows that, for the most part, these regions are distinct from those implicated in dyslexia, but there are some overlaps, notably 1p36, 2q22–35, 3p12–q13, 4q12–13, 6p21–22, 6q12–14, 13q22–33, and 15q15–21 (Germano et al., 2010).

Because the regions are very large and contain many genes, it is not clear if these findings reflect **pleiotropy** – the same gene contributes to both disorders – or coincidence – distinct genes for the different disorders reside in the same location. One study directly addressed the issue of pleiotropy by performing a bivariate linkage scan for phenotypes of both disorders in a sample of sibs selected for reading disability (Gayán et al., 2005). A locus on chromosome 14q32 provided evidence for this effect. Another study addressed this issue in reverse – reading ability was studied in a sib sample ascertained for ADHD (Loo et al., 2004). Four regions of suggestive linkage were identified, two of which overlapped with dyslexia signals on chromosomes 16p and 17q that had provided suggestive signals in their previous linkage analysis for ADHD (Fisher et al., 2002b).

The dopamine receptors, particularly DRD4 on chromosome 11p15.5, have been extensively studied as candidates for ADHD. Dopamine receptors have also been studied in dyslexia, but although association of DRD1 with inattentive symptoms in a cohort with dyslexia was observed (Luca et al., 2007), no associations, or sequence alterations were observed for dyslexia and multiple dopamine receptor genes or the dopamine transporter gene even in cohorts that provided evidence for linkage to regions containing dopamine receptor genes (Nopola-Hemmi et al., 2001; Marino et al., 2003; Hsiung et al., 2004; Luca et al., 2007). Therefore, it is unlikely that the DRD genes play a major role in dyslexia.

### Speech sound disorder

Speech sound disorder is a childhood disorder that affects the ability to acquire speech that is easily understood. Children with SSD may distort, substitute, omit, or insert speech sounds, arriving at speech output that differs in various ways from the adult target forms (Pennington and Bishop, 2009). Examples of distortion and substitution errors are realizing the /s/ sound with a fronted tongue posture known as a frontal lisp or the /k/ sound as a [t] sound (“tat” for “cat”). Omissions and insertions represent phonologically based speech errors. Examples of omissions are reduced consonant clusters (“top” for “stop”) or omitted final consonants (“hou” for “house”), even though the omitted consonants are produced correctly in other contexts such as single consonants or word-initial position. A common example of insertion is an inserted schwa sound ([ǝ]) into a consonant cluster to create consonant-vowel-consonant-vowel pattern (e.g., “balue” for “blue”), which is less complex in terms of speech production. Epidemiologic and twin studies provided evidence for a genetic contribution to SSD (Lewis and Thompson, 1992; Felsenfeld and Plomin, 1997; Bishop, 2002). Although linkage analyses have been performed in SSD, these have largely been restricted to evaluations of candidate loci implicated in other developmental disorders such as dyslexia, autism, and Prader–Willi syndrome, based on the rationale that disordered speech is frequently comorbid in these disorders (Stein et al., 2004, 2006; Smith et al., 2005; Miscimarra et al., 2007). These studies yielded suggestive evidence for shared gene locations on 1p36–p34, 3p14.2–q13.32 and 6p22, and 15q14–q21. One notable exception to the candidate region approach was a genome wide linkage analysis in a family with a particularly severe form of verbal and oral motor apraxia (i.e., affecting both speech and non-speech oral movements) in the presence of impaired language comprehension and formulation, cognitive deficits, and differences in brain structures (Fisher et al., 1998); this analysis identified a mutation in *FOXP2* as the cause for the autosomal dominant disorder (Lai et al., 2001). *FOXP2* mutations account for only a small proportion of non-syndromic forms of apraxic speech, referred to as childhood apraxia of speech (CAS; MacDermot et al., 2005; Laffin et al., 2012) and have not been found in other forms of SSD or in LI (Meaburn et al., 2002; Newbury et al., 2002; O’Brien et al., 2003), a disorder that is often comorbid with SSD. Intriguingly, there are suggestive linkage signals for dyslexia on chromosome 7q32, but no mutations in *FOXP2* were found in dyslexic individuals from the six families that contributed to the linkage signal (Kaminen et al., 2003). It is important to note that *FOXP2*-related reading impairment would not meet the criteria for dyslexia because the syndrome includes cognitive impairment. In a carefully phenotyped dyslexia subject set without evidence for LI or SSD we observed associations of *FOXP2* and non-word repetition, a measure of phonological memory, and rapid alternating place of articulation (the /pataka/ task), raising the possibility of this gene’s influence on component phenotypes shared by multiple developmental disorders of language (Peter et al., 2011b).

Two recent studies screened genome-wide markers in individuals with CAS. In a sample of 24 unrelated individuals with a CAS diagnosis, 12 showed evidence of deleted or duplicated genetic material on 10 different chromosomes (Laffin et al., 2012). The authors interpret their findings as supportive of a heterogeneous CAS etiology. Our research team explored the genetic basis of SSD using a motor sequencing deficit as a marker of affectation status in a multigenerational family where two of the children had a CAS diagnosis. Although the family was too small to provide statistical evidence for linkage, four regions of interest were revealed in a genome wide microsatellite study, two of which overlapped with previously reported regions for dyslexia, 6p21 for a composite measure of rapid naming (König et al., 2011) and 7q32 for categorical dyslexia diagnosis (Kaminen et al., 2003) and non-word and irregular word spelling (Bates et al., 2007), and a third locus at 7q36 borders on the region containing *CNTNAP2*, a gene that interacts with FOXP2 and is thought to affect the component traits of LI (Vernes et al., 2008). More recently, we showed that the CAS phenotype in multigenerational families was characterized not only by deficits in sequential processing at the level of alternating oral motor movements, which is consistent with the traditional CAS definition as a motor programming disorder, but also by deficits in sequential hand movements, indicating a systemic motor deficit, and by sequential professing deficits at the level of phoneme sequences during word and non-word imitation as well as during non-word decoding and spelling (Peter et al., 2012). This finding may have relevance for dyslexia, as decoding unfamiliar words and spelling words from long-term memory require high loads of sequential processing.

A review of the SSD/reading impairment comorbidity literature reveals that certain subgroups of children with SSD are more likely to also struggle with learning to read, namely children who also have difficulty with comprehending and/or formulating oral language (Sices et al., 2007), children with deficits in phonological processing (Bird et al., 1995), children whose SSD persists into the school years (Nathan et al., 2004), and children with CAS (Lewis et al., 2004).

### Language impairment

Language impairment, also referred to as specific language impairment (SLI), is a disorder that manifests as deficits in comprehension and/or expression of language in the presence of typical development in other areas. Syntactic awareness and vocabulary can also be deficient and many children with LI begin to talk later than their typically developing peers and produce a smaller number of different words during conversation (Watkins et al., 1995). Low scores in three types of tasks have been proposed as clinical markers of LI. Several studies showed that children with LI struggle with non-word imitation tasks, which tap into phonological processing and short-term memory, significantly more than typical peers (Dollaghan and Campbell, 1998; Weismer et al., 2000). Sentence imitation tasks tap into short-term memory, semantic processing, and syntactic competence, and children with LI imitate sentences with significantly lower accuracy, compared to typical peers (Stokes et al., 2006; Conti-Ramsden et al., 2007). English has finiteness markers such as the third person singular present tense verb suffix /s, z, ǝz/ (“she gives”). Children with LI have a higher tolerance for missing finite markers when making grammaticality judgments and produce more finite marker errors than typical peers (Rice et al., 1995; Rice and Wexler, 1996). Consequently, LI differs from dyslexia in that dyslexia is defined with respect to written language and especially at the single word level, whereas LI refers to difficulties with spoken language and especially with respect to the interactions of multiple words in terms of syntactic frames and semantic relationships. Downstream effects of dyslexia may include an impoverished vocabulary due to diminished exposure to text as a source of world knowledge, whereas LI may influence reading comprehension due to deficits in deriving syntactic and semantic information from written words. As in the cases of dyslexia, SSD, and ADHD, there is substantial evidence that LI has a genetic etiology (Bishop et al., 1995; Tallal et al., 2001; Hayiou-Thomas, 2008) and several susceptibility loci have been identified, on chromosomes 6, 7, 12, 13, and 17 (Villanueva et al., 2011), 12 (Addis et al., 2010), 13q (Bartlett et al., 2002), 16q (SLI1), and 19q (SLI2), respectively (Bartlett et al., 2002; Consortium, 2002). The locus on chromosome 16 contains two LI candidate genes, *ATP2C2* and *CMIP* (Newbury et al., 2009), and *CNTNAP2* on chromosome 7 has also been shown to influence LI (Vernes et al., 2008). LI and SSD co-occur more frequently than expected under random conditions (Shriberg et al., 1999; Pennington and Bishop, 2009) and children with SSD and concomitant LI are at a higher risk for developing reading disorders compared with children with isolated SSD (Sices et al., 2007). In a sample of children with LI, evidence for linkage to candidate loci for measures of speech and reading ability was found (Rice et al., 2009). This finding may indicate that speech, language, and reading disorders have some shared genetic etiologies that involve multiple genes.

### Co-occurrence of ADHD, SSD, LI, and dyslexia

Definitional imprecision complicates estimation of the frequency with which ADHD, SSD, LI, and dyslexia co-occur (Gilger et al., 1992; Cohen et al., 2000; Peterson et al., 2007; Pennington and Bishop, 2009). Impairments in phonological short-term memory, as assessed by one or more non-word repetition tasks, characterize dyslexia, LI and SSD, and there is evidence for linkage of this phenotypic trait to loci associated with each of these. This observation illustrates the likelihood that there are probably multiple pathways to even the phenotypic components of these disorders. In LI, evidence for linkage of non-word imitation to both SLI1 and SLI2 has been repeatedly obtained (Consortium, 2002; Velayos-Baeza et al., 2007; Falcaro et al., 2008; Newbury et al., 2009), whereas for SSD linkage was observed to DYX2 on chromosome 6p21 (Smith et al., 2005). More detailed information about comorbidity estimates in communication disorders and associated traits can be found in recent reviews (Peterson et al., 2009; Snowling and Hulme, 2012).

## Candidate Genes

A review of dyslexia candidate regions and knowledge of suspected genetic etiologies in disorders frequently also characterized by difficulty with learning to read and spell can provide a meaningful platform from which to describe actual candidate genes for dyslexia. At least fourteen candidate genes have been proposed for dyslexia, but to date some have little supportive evidence. Within the three most-replicated dyslexia loci, four candidate genes for dyslexia have been identified: *DYX1C1* in DYX1 on chromosome 15q21 (Taipale et al., 2003b), *DCDC2* (Meng et al., 2005b) and *KIAA0319* (Cope et al., 2005a) in DYX2 on chromosome 6p21, and *ROBO1* in DYX5 on chromosome 3p12–q12 (Hannula-Jouppi et al., 2005).

*The DYX1 locus*. *DYX1C1* (dyslexia susceptibility 1 candidate 1; MIM 608706) was identified in a study of a family with a **structural chromosomal rearrangement**, t(2;15; q11;q21), that **segregated** with mild intellectual impairment and/or dyslexia (Nopola-Hemmi et al., 2000). The breakpoint on chromosome 15 was 15 Mb distal to the DYX1 linkage region but was found to disrupt a gene of unknown function (Taipale et al., 2003a). The gene was colloquially called EKN1 but later renamed dyslexia susceptibility 1 candidate 1. DYX1C1 is a widely expressed 420-amino acid cytoplasmic and nuclear protein that contains three tetratricopeptide repeat (TPR) domains thought to be involved in protein–protein interactions, and the breakpoint of the translocation was within a TPR domain-coding region (Taipale et al., 2003a).

In an association study of Finnish individuals with and without dyslexia, the minor alleles of two **single nucleotide polymorphisms (SNPs)** in *DYX1C1* were suggested to be of functional significance: −3G > A (rs3743205), because it creates an ELK-1 **transcription** binding site very near the **translation** initiation site, and 1249G > T, because it truncates the protein by four amino acids (Taipale et al., 2003a). The design of this original association study was flawed in that the cases and controls were related. Multiple groups have investigated the relationship of alleles of both polymorphisms to dyslexia phenotypes. Association of one of these putative functional alleles with dyslexia was found in a study of Chinese children (Lim et al., 2011) and with short-term memory in Italian dyslexics (Marino et al., 2007), but other groups found only modest association with the **major alleles** (Scerri et al., 2004; Wigg et al., 2004; Brkanac et al., 2007), and yet others failed to detect an association with any of the alleles (Bellini et al., 2005; Cope et al., 2005b; Marino et al., 2005; Meng et al., 2005a; Bates et al., 2010), suggesting that none of these SNPs is the susceptibility determinant. However, expression of a construct containing the −3A allele of rs3743205 was lower than one containing the −3G allele when **transfected** into a neuroblastoma cell line (Tapia-Páez et al., 2008). Two other SNPs in the 5′ **untranslated region (UTR)** of DYX1C1 have been reported to associate with dyslexia both singly and in a **haplotype** with rs3743205 (Dahdouh et al., 2009) and to be recognition sites for **transcription factors** TFII-I and Sp1: TFII-1 toboth alleles of rs3743205 and Sp1 to rs16787 (−10310C > A) and rs12899331 (−10567T > C). The transcription factors TFII-I, poly (ADP-ribose) polymerase 1 (PARP1), and splicing factor proline/glutamine-rich (SFPQ) bind to the −3A-containing rs3743205 site to form a complex with DYX1C1 (Tapia-Páez et al., 2008). Therefore, allelic differences in the 5′ UTR of *DYX1C1* may affect its regulation.

The often-reported difference between the prevalence of dyslexia in males and females raises the possibility that hormones may play a role in its pathogenesis. There is some evidence that estrogen replacement does improve reading ability in postmenopausal women (Shaywitz et al., 2003). It has been shown that the rat **homologue** Dxy1c1 interacts with estrogen receptors alpha and beta in primary rat neuronal cells (Massinen et al., 2009). Recent studies in a human neuroblastoma cell line found that estrogen receptor beta and the transcription factor TFII-I are present simultaneously on a region near the *DYX1C1* transcriptional start site. DYX1C1 expression is enhanced by 17β-estradiol (E2) and alleles of SNP rs3743205 affect the regulation of DYX1C1 by estrogen receptor beta (Tammimies et al., 2012a). These observations could imply involvement of estrogen signaling and neuronal migration in dyslexia. Another interesting observation was that when the −3G allele of rs3743205 (that lies within a **CpG island**) was **methylated**, it had a drastic effect on DYX1C1 transcription (Tammimies et al., 2012a). Therefore, it was postulated that this polymorphism might have a functional effect through an estrogen-signaling pathway and/or through methylation-dependent gene activity status, an epigenetic mechanism.

*The DYX2 locus* proved more complex, as it contains two candidate dyslexia susceptibility genes, *KIAA0319* in the proximal portion and *DCDC2* (doublecortin domain-containing protein 2; MIM 605755) approximately 200 kb distal. These genes were identified by different groups using similar association-based strategies to narrow the linkage region and detect potentially causative variants (Francks et al., 2004; Meng et al., 2005b). Supportive evidence for involvement of *KIAA0319* had been obtained using some of the same subjects (Kaplan et al., 2002; Deffenbacher et al., 2004) and in an independent sample (Cope et al., 2005a; Harold et al., 2006). Variants in the 5′ *UTR* of the gene, where the **promoter** resides, provide the strongest evidence for association, but results are not always significant and not always in the same direction (Couto et al., 2010; Elbert et al., 2011). The minor allele of SNP rs9461045 in the 5′ UTR was shown to reduce expression in neuronal cells (Dennis et al., 2009). This SNP creates a nuclear protein-binding site for a transcriptional silencer, OCT-1, and **RNAi knockdown** of *OCT-1* in a neuronal cell line restores *KIAA0319* expression from the risk haplotype. RNA interference (RNAi) studies subvert a naturally occurring process of post-transcriptional gene silencing (Carthew and Sontheimer, 2009; Ketting, 2011). In this procedure, plasmids containing short double stranded RNA molecules complementary to part of the coding sequence of a gene are synthesized. These small molecules form a hairpin structure (shRNA) that specifically binds to the normal message and prevents its translation into protein.

**Splice variants**, protein structure, post-transcriptional modification, and interaction partners of KIAA0319 have been extensively studied. A major transcript is widely expressed in brain, particularly in cerebellum, cerebral cortex, putamen, amygdala, and hippocampus (Velayos-Baeza et al., 2007, 2008, 2010; Levecque et al., 2009). KIAA0319 has a transmembrane domain, but undergoes intramembrane cleavage, after which the soluble cytoplasmic domain translocates to the nucleus and accumulates in nucleoli, suggesting that it may function in a signaling pathway and have a role in the regulation of gene expression (Velayos-Baeza et al., 2010).

Because no causative coding variant has yet been found in *KIAA0319* (or in the other candidate genes), it is reasonable to postulate that changes in regulation of the gene(s) may be the pathogenic mechanisms. Cell- and developmental stage-specific regulation of gene transcription is a complex process involving epigenetic modification of DNA. One such epigenetic tag is acetylation of **histones** that causes relaxation of condensed **chromatin**, comprised of protein and DNA, so that it is accessible to the transcription machinery (Vaissière et al., 2008; Wang et al., 2009). In a retinoblastoma cell line that shares characteristics with neuronal stem cells, the DYX2 region was mapped by a multistep procedure (ChIP-chip) to detect acetylated histones (Couto et al., 2010). In the first part of the procedure (ChIP), chromatin is treated to bind the protein of interest to the DNA (cross-linking) to prevent detachment. The cells are then lysed, the specific protein-DNA complexes are immunoprecipitated with antibody to the protein, and proteins are removed. In the next part of the procedure (chip), the DNA sequence is determined by hybridization to genomic arrays called chips. This approach identified a cluster of acetylated histones that mapped to 2.7 kb within the 5′ region of *KIAA0319*. Multiple SNPs previously associated with differential expression of the gene were contained in this small segment and six additional ones were identified in a 22 kb haplotype block that encompassed this region (Couto et al., 2010). One or more of these polymorphisms might alter transcriptional regulation of *KIAA0319* and thus play a role in dyslexia. For example, as assessed by tandem mass spectrometry of KIAA0319 transcripts in neuroblastoma and lymphoblastoid cell lines, expression was consistently lower from the allele bearing the putative risk haplotype suggesting that it is frequently inherited together with a regulatory sequence variant that negatively affects transcription (Paracchini et al., 2006).

Association of dyslexia with SNPs in the 5′ *UTR* of the *DCDC2* gene has also been found (Deffenbacher et al., 2004; Meng et al., 2005b). Two polymorphisms in *DCDC2* are potential functional variants, a 2445 bp deletion in **intron** 2 and a compound short tandem repeat within the deleted region (BV677278; Meng et al., 2005b). Approximately 17% of dyslexic subjects in a US sample carried the deleted allele and, what seemed even more tantalizing, all subjects with the deleted allele had dyslexia. In addition alleles of BV677278 were recently shown to affect transcription through an enhancer mechanism (Meng et al., 2011). In an Italian sample set the deletion appeared to associate with severity of impairment on quantitative traits (Marino et al., 2012). However evidence for a causative role of one or both of these polymorphisms has been elusive in other subject samples. We did not observe association of *DCDC2* alleles with dyslexia in our multigenerational US sample, although there was a slight tendency for the intronic deletion to be associated with worse performance on some quantitative measures of dyslexia in the probands, but not in their parents (Brkanac et al., 2007). The German sample that had provided supportive evidence for the DYX2 region (Schumacher et al., 2006a) did not show transmission deviation for either the deletion or the complex STR polymorphism (Ludwig et al., 2008). As is increasingly apparent from burgeoning exome sequencing projects on numerous disorders, the exome of an individual contains 10,000–15,000 variants, including premature stop codons, and these are often phenotypically silent (Ng et al., 2008).

*The DYX5 locus*. The *ROBO1* gene that encodes an axon guidance receptor was found by identifying the breakpoints of a translocation, t(3;8; p12;q11), in a man with dyslexia and infertility (Hannula-Jouppi et al., 2005). The breakpoint on chromosome 3, in the DYX5 and SSD locus (Nopola-Hemmi et al., 2001; Stein et al., 2004), fell in the first intron of the gene. Interpretation of the family history is complicated by the presence of dyslexia in one sibling and intellectual impairment in his two other siblings, none of which carries the translocation. Because the Drosophila melanogaster homolog of *ROBO1* is involved in formation of neural connections between the left and right brain (Kidd et al., 1998) and because ROBO1 activity was reduced in 19 dyslexic members of the large Finnish family that had provided linkage to this region (Nopola-Hemmi et al., 2001), the authors hypothesized that dyslexia in rare families may be caused by partial **haploinsufficiency** of the gene. SNPs rs6803202 and rs4535189 in *ROBO1*, that have not been shown to have a functional effect, associate significantly with phonological memory but not reading or spelling in an unselected population of twins and their siblings (Bates et al., 2011 #3602). These results led the authors to propose that this gene is a candidate for LI but not dyslexia. Knockout of the mouse homolog, *Robo1*, prevents axons from crossing the corpus callosum and is lethal at birth (Andrews et al., 2006). It was hypothesized that poor axonal crossing might have an effect on binaural hearing in individuals in the Finnish family who carried the risk *ROBO1* haplotype (Lamminmäki et al., 2012). Frequency-tagged magnetoencephalographic (MEG) tests of binaural suppression (Kaneko et al., 2003), an indirect assessment of axonal crossing in auditory pathways, did reveal defective interaural interaction.

*Other Proposed Candidate Genes*. Several other genes deserve mention, but await corroboration before they can be added to the list of candidate genes for dyslexia susceptibility. *DOCK4* in the AUTS1 locus on chromosome 7q31.1 was proposed as a dyslexia candidate gene in a study of autism. In one Dutch family, autistic siblings carried a maternally inherited microdeletion that created a fusion of *DOCK4* and another gene, *IMMP2L*, and a paternally-inherited microdeletion that disrupted a gene in the contact in associated protein family (*CNTNAP5*) that has been implicated in autism (Pagnamenta et al., 2010). Another sibling did not have a diagnosis of autism but had a reading impairment as did the mother, one of her brothers, and two of his children. One of the autistic siblings and two other siblings of the mother also carried the microdeletion but were not reading impaired. Given the co-occurrence of the *DOCK4* disruption and reading impairment in six of nine people in the family, a panel of 606 unrelated individuals with dyslexia was evaluated. This study detected a *DOCK4* microdeletion in a boy who inherited it from his father who had slow reading speed; his sister who had no reading difficulties did not carry the microdeletion.

By **fluorescence** **in situ hybridization (FISH)** and SNP microarray analyses, a small deletion on chromosome region 21q22.3 was found in all four reading disabled members of a family (Poelmans et al., 2009). The deletion involved four brain-expressed genes, *PCNT*, *DIP2A*, *S100B*, and *PRMT2*. It is not known which, if any, of these genes is responsible for the phenotype, but arguments have been put forward in favor of *DIP2A*, because it encodes a protein that interacts with the glutamate receptor (Yu et al., 2001) and might be involved in synaptic plasticity, and *PCNT*, because it encodes a protein required for the assembly of the primary cilium (Jurczyk et al., 2004). Mutations in PCNT cause Seckel syndrome, an autosomal recessive disorder of marked microcephaly and dwarfism (Griffith et al., 2008).

Another potential candidate gene in the DYX1 locus has been proposed. The complex promoter region of *CYP19A1* was disrupted by the breakpoint of a translocation, t(2;15; p12;q21), carried by four people in a Finnish family, one of whom was dyslexic (Nopola-Hemmi et al., 2000; Anthoni et al., 2012). *CYP19A1* codes for aromatase, an enzyme that converts androgens into estrogens (Boon et al., 2010). This is a tantalizing finding in light of the previously mentioned hypothesis that sex hormones may play a role in development of dyslexia. Some evidence for association of SNP haplotypes in CYP19A1 with dyslexia defined categorically was found in Finnish and US dyslexia cohorts, but not in a German one (Anthoni et al., 2012). These same haplotypes also showed association with SSD, a disorder with evidence of linkage to the chromosome 15q21 region. In the dyslexia cohorts, less significant associations were observed for quantitative traits, including oral motor skill and nonsense word repetition, traits that characterize SSD. Two dyslexia-associated SNPs that flank the brain-specific promoter of *CYP19A1* affected the binding of several transcription factors that also bind to *DYX1C1* (Tapia-Páez et al., 2008). Patterns of brain expression of *CYP19A1* paralleled those of *DYX1C1*, *DCDC2*, *KIAA0319*, *ROBO1*, and *C2ORF3* and were most strongly correlated with *ROBO1* and *DYX1C1* (Anthoni et al., 2012). Finally, aromatase knockout mice had evidence of cortical disorganization, including increased neuronal density and occasional **heterotopias**.

Linkage studies in Finnish families implicated loci on chromosomes 2 and 7 in dyslexia (Kaminen et al., 2003). Through association studies in Finnish and German subject samples, two candidate genes, *C2Orf3* and *MRPL19*, were proposed for DYX3 on 2p16–p15 (Anthoni et al., 2007). *C2Orf3* and *MRPL19* RNA was decreased in heterozygous carriers of a risk haplotype. However, little is known about the function of the proteins coded by them, and large association studies of English and Australian children found no support for involvement of this locus in dyslexia (Paracchini et al., 2011; Scerri et al., 2011).

Spurred by a supportive linkage signal for non-word reading in Australian twins for one marker in the chromosome 7q22–q34 locus (Bates et al., 2007), fine mapping, and association studies were done in German and Finnish sample sets (Kaminen et al., 2003). No support for linkage to this region was found in the German sample, but results in the Finnish sample set, expanded from the one used originally to identify the peak, considerably narrowed the region of interest. In this narrowed region, overlapping haplotypes within the diacylglycerol kinase gene *DGKI* were detected in both samples, but only the Finnish association remained significant after Bonferroni correction. DGKI can modulate receptor-dependent responses in processes such as synaptic transmission synaptic transmission (Merida et al., 2008), and thus deserves consideration as a susceptibility gene for dyslexia.

MC5R, DYM, and NEDD4L have been proposed as candidate genes for the dyslexia locus on chromosome 18 (Scerri et al., 2010). These and other genes and loci that showed association with dyslexia in single studies continue to be reported but require confirmation. Many of the observed associations are driven by the subsets of most severely affected individuals.

As mentioned above, the distinction between the various disorders of written language is not discrete and phonological memory impairment characterizes them all. The phenotypic overlap might reflect shared genetic contributions. *GRIN2B* is a candidate gene for the chromosome 12p12 locus for phonological memory identified in our American dyslexia sample (Brkanac et al., 2008); the variant rs1012586 was significantly associated with phonological memory in a German dyslexia sample (Ludwig et al., 2010). Several candidate genes for LI, *CMIP*, *ATP2C2*, and *CNTNAP2*, are also associated with phonological memory (Newbury et al., 2009; Peter et al., 2011b) and variants in *CMIP* (e.g., rs6564903) may affect single word reading and spelling (Newbury et al., 2011; Scerri et al., 2011).

**Copy number variation (CNV)**, a form of DNA derangement increasingly implicated in developmental disorders (Morrow, 2010; Coe et al., 2012) deserves mention. We investigated the role of CNVs in dyslexia, sporadic autism, and intellectual impairment. We observed a gradient of frequency of these changes increasing with more severe intellectual impairment (Girirajan et al., 2011). In more than 350 children with dyslexia, there was essentially no difference in large CNV burden compared to controls.

The potential involvement of dyslexia candidate genes with reading skills in the general population has also been explored. In a longitudinal study of a cohort of English children, associations were observed between *DCDC2* and dyslexia, and between *CMIP* and *KIAA0319* and single-word reading and spelling across the ability range; significance was increased by inclusion of individuals with comorbid LI or ADHD (Scerri et al., 2011). In contrast, no support for involvement of *MRPL19/C2ORF3* in reading abilities was obtained and results for *DYX1C1* were weak. The major allele of rs3743205 (−3G allele) showed a trend of association with poor reading performance, and the minor allele of rs685935 showed a trend of association with poor spelling performance. An association study of markers in *DYX1C1*, MRPL19/*C2ORF3*, *KIAA0319*, and *DCDC2* with quantitative performance on reading comprehension and ability to correct spelling mistakes or supply missing words was performed in a longitudinal cohort of 520 Australian individuals (Paracchini et al., 2011). This study found that the same three-marker haplotype in *KIAA0319* and the minor allele of SNP rs2143340 that was associated with dyslexia and reduced expression (Paracchini et al., 2008) was also associated with poorer reading and spelling performance in an unselected population and the results were strengthened by adjustment for IQ. Finally, a missense mutation (Val91Ile) in *DYX1C1* was suggested to be functionally related to reading, spelling, and short-term memory in a large set of adolescents not selected for dyslexia (Bates et al., 2010) but this finding has not yet been replicated.

## Relationships between Candidate Genes and Brain Structure

### A proposed causative role of brain pathology in dyslexia

For more than 40 years, the existence of anatomic brain correlates of dyslexia have been postulated. From the late 1960s to mid 1980s, abnormalities were detected in autopsied brains from individuals with histories of reading impairment (Drake, 1968; Galaburda and Kemper, 1978; Galaburda et al., 1985). The first case described by Galaburda demonstrated delayed speech development, impairments in semantic and mathematical ability, and epilepsy (Galaburda and Kemper, 1978). Abnormalities were found predominantly in the left hemisphere and included an area of polymicrogyria in the left planum temporale and posterior portion of the transverse gyrus of Heschl, disordered cortical layering in the cingulate gyrus and rostral insula, and minute foci of dysplasia in the parietal, occipital, and temporal lobes. Three additional men were then studied (Galaburda et al., 1985). In addition to reading and spelling impairments, one of these subjects had a very low IQ, one was reported to have “notable” language difficulties, and one had delayed speech acquisition. Neuronal **ectopias** and architectonic dysplasias located mainly in perisylvian regions and affecting predominantly the left hemisphere were found in all three cases. The ectopias are thought to result from abnormal migration of neurons during brain development. The early postmortem studies also suggested that the left and right planum temporale regions were equal in size in the brains of individuals with dyslexia, whereas in typical individuals, the right planum temporale is smaller than its left counterpart (Livingstone et al., 1991; Galaburda et al., 1994). More recent research suggests, however, that this lack of asymmetry is seen more frequently when poor reading ability coincides with poor language ability, compared to isolated poor reading ability (Leonard and Eckert, 2008).

During embryonic brain development, neuronal progenitors go through a proliferation phase, differentiate into postmitotic neurons, and then migrate to specific locations. Defects in neuronal migration can be caused by single gene mutations and often have severe developmental consequences, including cognitive impairment and intractable epilepsy (Liu, 2011). However, there is a wide range of manifestations and severity, representative of the genetic heterogeneity. One of these disease subtypes is periventricular nodular heterotopia (PVNH). A study of 10 individuals with MRI-documented PVNH found that all had current difficulty with phonology, reading, and spelling and most had a history of problems with reading, but only four had IQ measurements more than a standard deviation below the mean (Chang et al., 2005). A subsequent study found that individuals with dyslexia had more severe phonological impairment that those with PVNH, but both groups had impaired rapid naming, related to reading fluency (Chang et al., 2007). In those with PVNH, diffusion tensor imaging, an MRI-based imaging method to characterize fiber tract anatomy, found that severity of rapid naming difficulty correlated directly with degree of focal disruptions in white matter microstructure and organization in the vicinity of gray matter nodules. The authors proposed that the findings in PVNH, by extension, support the functional association of ectopias and dyslexia and suggest a biological mechanism for the behavioral defect. However, some caution should be exercised in extrapolating directly from PVNH to dyslexia with respect to this mechanism. Widespread neuronal ectopias do not predict existence or pattern of reading impairment (Reinstein et al., 2012) and may be associated with non-verbal learning impairment (McCann et al., 2008).

### Evidence for involvement of dyslexia candidate genes in brain pathology

It is notable that 10 candidate dyslexia genes are members of a proposed molecular network involved in neuronal migration and neurite outgrowth (*ROBO1*, *KIAA0319*, *KIAA0319L*, *S100B*, *DOCK4*, *FMR1*, *DIP2A*, *GTF2I*, *DYX1C1*, and *DCDC2*; Poelmans et al., 2011) and axon guidance (Hannula-Jouppi et al., 2005; Poon et al., 2011). A role in neuronal migration during brain development has been suggested for multiple dyslexia candidate genes. DYX1C1 is expressed in developing rat forebrain as well as in adult brain (Rosen et al., 2007). To investigate the importance of DYX1C1 protein on brain development, RNAi knockdown experiments were performed (Wang et al., 2006; Rosen et al., 2007). When short DNA pieces containing shRNA specific for the rat homolog (*Dyx1c1*) of human DYX1C1 were injected into rat brains *in utero*, the orderly migration of transfected neurons was disrupted, either traveling too short or too far a distance from the ventricular zone (Wang et al., 2006; Rosen et al., 2007; Currier et al., 2011). In some animals, ectopias were seen in the molecular layer that were similar to the abnormalities previously reported in brains of people with a history of reading impairments. Because GABAergic neurons that did not contain the shRNA were also present in the ectopias, it is postulated that they were affected by the cells that did contain the shRNA (Currier et al., 2011). Knockdown of *DYX1C1* was also associated with auditory processing and spatial learning deficits (Threlkeld et al., 2007).

Working memory deficits have been documented in dyslexic individuals (Swanson and Berninger, 1995; Swanson and Siegel, 2001; Brooks et al., 2011; Martinez Perez et al., 2012) and an association between *DYX1C1* variants and memory deficits has been observed (Marino et al., 2007). Rats treated *in utero* with RNAi of *Dyx1c1* exhibited a subtle, but significant and persistent impairment in working memory as assessed by a radial water maze task (Szalkowski et al., 2011). Recently, gene expression and protein interaction profiling in a human neuroblastoma cell line revealed that DYX1C1 can modulate the expression of nervous system development and neuronal migration genes such as RELN and associate with a number of cytoskeletal proteins (Tammimies et al., 2012b).

Fluorescence *in situ* hybridization revealed a pattern of expression in fetal mouse and human brains at various stages of development that suggested KIAA0319 plays a role in neuronal migration during formation of the cerebral cortex (Paracchini et al., 2006). As was seen for *DYX1C1*, *in utero* RNAi knockdown in rat brain of the rat homolog, *Kiaa0319*, altered neuronal morphology, and disrupted the normal outward migration of neurons from the ventricular zone toward the cortical plate (Paracchini et al., 2006; Peschansky et al., 2010); overexpression did not result in periventricular heterotopias. The ectopias contained both transfected and non-transfected neurons, suggesting that the mechanism of migration disruption is both cell autonomous and non-cell autonomous. In addition, neurons transfected with Kiaa0319 shRNA exhibited apical dendrite hypertrophy, showing that Kiaa0319 is involved in growth and differentiation of dendrites as well as neuronal migration (Peschansky et al., 2010). Embryonic RNAi-knockdown of rodent *Kiaa0319* also results in impaired acoustic discrimination as assessed by a modified prepulse inhibition paradigm (Szalkowski et al., 2012). Furthermore, in the subset of animals that exhibited impaired spatial learning abilities on Morris and radial arm water maze testing, hippocampal dysplasia was seen. However, in contrast to *DYX1C1*, no significant impairment in working memory was detected in these animals (Szalkowski et al., 2012).

DCDC2 protein belongs to the doublecortin domain containing family of genes. Other members of that family have been shown to bind to and stabilize assembly of microtubules, and mutations in mice interfere with neurogenesis, dendrite formation, and neuronal migration (Corbo et al., 2002; Kerjan et al., 2009; Pramparo et al., 2010). Therefore, it has been speculated that the effect of abnormal DCDC2 in dyslexia might be mediated through disruption of microtubule-mediated movement of proteins and cell migration. Although the specific type of gene change in *DCDC2* that is responsible for its effect on reading remains unknown, under the assumption that the pathogenic change would decrease the activity of the protein, RNAi studies were performed. As was seen for *DYX1C1*, knockdown of the rat *Dcdc2* message resulted in neuronal migration anomalies (Meng et al., 2005b; Burbridge et al., 2008). Some neurons migrated only a short distance from the ventricular zone resulting in heterotopias, and others migrated past their expected lamina. Co-transfection of *Dcdc2* shRNA and a human *DCDC2* overexpression construct rescued the periventricular heterotopia phenotype, but did not prevent transfected neurons from migrating too far (Burbridge et al., 2008). Overexpression of either rat *Dcdc2* or human *DCDC2* did not cause any malformations.

The primary cilium is a solitary organelle in most cells that is involved in signaling pathways during development and in homeostasis (Satir et al., 2010). It is comprised of microtubules. A role for DCDC2 in the structure and function of primary cilia was suggested by studies in cultured rat hippocampal neurons (Massinen et al., 2011). In the presence of overexpressed DCDC2, the primary cilia grew longer. Overexpression of human DCDC2 or the *Caenorhabditis elegans* homolog in *C. elegans* caused ectopic branching at the cell soma and dendrites of ciliated neurons. The ciliopathies are a phenotypically varied group of disorders in humans, some of which are characterized by abnormal development of the central nervous system (Ansley et al., 2003; Dixon-Salazar et al., 2004).

Studies in a knockout mouse model showed that complete loss of Dcdc2 protein was not required for normal brain development (Wang et al., 2011); neuronal migration, neocortical lamination, neuronal ciliogenesis, and dendritic differentiation were all essentially normal. But knockdown of other doublecortin proteins by RNAi resulted in more severe neuronal migration and dendritic abnormalities in *Dcdc2* knockout mice than in wild-type mice. These results indicate that in mice Dcdc2 probably has partial functional redundancy with other doublecortin family members. In contrast to the knockout mice whose performance was indistinguishable from wild-type, mice carrying a heterozygous deletion of *Dcdc2* **exon** 2 were impaired in visual long-term memory, as measured by a test of novel object recognition, and had decreased performance efficiency on maze testing although they retained the ability to learn the task (Gabel et al., 2011). Alteration of visuo-spatial perception is one proposed etiology for dyslexia (Smith-Spark et al., 2003; Vidyasagar and Pammer, 2010).

These studies show that substantial suppression of the dyslexia candidate genes in animals produces brain abnormalities, some of which are similar to what was seen in the postmortem anatomic studies of Galaburda and others. However, no variants predicted to have such a severe effect on the function of the genes have yet been found in dyslexic individuals.

## Relationships between Candidate Genes and Brain Function

A comprehensive review of the research over the past three decades on the brain basis for dyslexia and the various imaging tools used to study different aspects of brain structures and functions is beyond the scope of this article; such information is presented in multiple recent publications (Richards et al., 2007; Sandu et al., 2008; Siok et al., 2008; McCardle et al., 2011; Linkersdörfer et al., 2012; Raschle et al., 2012; Vandermosten et al., 2012). In addition to structural abnormalities documented by pathology and imaging (Darki et al., 2012), functional abnormalities (Linkersdörfer et al., 2012) have also been identified. Moreover, it is increasingly clear that genes are involved not only in neural migration early in brain development but also in the functioning of the brain throughout development. Genes play a role in regulating efficiency of glucose utilization (energy supplies; Roeske et al., 2011) and mRNA transcription and translation processes in individual neurons in different regions of the brain (Batshaw et al., 2007; Kandel et al., 2012). In addition, the brain continues to change across development (Linkersdörfer et al., 2012).

Increasingly research attention is being directed to these various roles of genes in brain development, brain structures, and brain functions (e.g., Richards et al., 2006; Darki et al., 2012; Pinel et al., 2012; Wilcke et al., 2012). Studies of the correlation of genetic polymorphisms with interindividual variability in brain activation and functional asymmetry in frontal and temporal cortices revealed that SNPs rs6980093 and rs7799109 in *FOXP2* were associated with variations of activation in the left frontal cortex (Pinel et al., 2012). In the three-gene cluster containing *KIAA0319*, rs17243157 was associated with asymmetry in functional activation of the superior temporal sulcus (STS). These results suggest that *FOXP2* and *KIAA0319* both play an important role in human language development. Their observed cortical effects mirror previous fMRI results in developmental language and reading disorders, and suggest that a continuum may exist between these pathologies and normal interindividual variability (Pinel et al., 2012). By fMRI a significant main effect was observed for the factor “genetic risk” in a temporo-parietal area involved in phonological processing and a significant interaction effect was observed between the factors “disorder” and “genetic risk” in activation of inferior frontal brain areas in dyslexia (Wilcke et al., 2012). This result hints at the role of *FOXP2* genetic variants in dyslexia-specific brain activation and demonstrates use of imaging genetics in dyslexia research. The neuropsychology, brain bases, and genetics research on related disorders of language development – dyslexia, LI, and SSD – and the relationships of the three disorders to each have been previously reviewed (Peterson et al., 2007).

## Epigenetics

A now substantial body of research has shown that brain structures and functions change over time in response to both genetic and environmental variables. Attention is increasingly being paid to the role of **epigenetic** influences on phenotypes and disorders. Epigenetic changes are erasable – that is they do not change the underlying DNA sequence. Some epigenetic tags are placed by methylation of DNA, acetylation of histones, or phosphorylation of proteins. In contrast to genetic influences that are passed from one generation to the next, epigenetic influences are transmitted from a cell to its progeny. Such epigenetic tags allow genes to be expressed at specific developmental stages and in specific tissues and some are involved in learning and memories. The localization and function of regulatory elements in the genome are being studied through the multisite consortium Encyclopedia of DNA Elements (ENCODE; Dunham et al., 2012).

The role of epigenetics is now being explored to investigate how environmental influences may alter genetic expression at both the brain and behavioral levels. The first step is to characterize the observable/measurable changes. The brain’s response to reading, writing, and math instruction has been recently reviewed (Berninger and Dunn, 2012). Interdisciplinary research is advancing knowledge of brain-genetics-and behavioral assessment/instructional research (Richards et al., 2006). This research team drew on neuroanatomical (Eckert and Leonard, 2000; Eckert et al., 2003, 2005 #3674), family genetics (Raskind et al., 2005; Igo et al., 2006; Rubenstein et al., 2011), functional imaging, and related intervention studies (Berninger and Richards, 2010) to provide a converging, cross-disciplinary model for a working memory architecture supporting written language learning. This architecture has a distinct brain signature for storing and processing phonological, orthographic, and morphological word forms and mapping them onto one another. Both biological phenotypes (brain structures and brain functions) were shown to differentiate those with and without dyslexia before but not after specific kinds of language instruction.

## Conclusion

Progress is being made in understanding the genetic and behavioral variability in learning to read. Clearly, there are compelling links among the findings from studies of genetic linkage and association, functions of candidate genes, brain structures and functions, and associated speech and language abilities. In part, the composite picture of reading disability that has emerged to date is complicated by methodologic issues such as evaluation of different traits in different participant samples. If dyslexia is heterogeneous, studying a sample of individuals from different families means studying a mixture of genetic etiologies. Continued progress will benefit from exploring the complex relationships between the genetic and behavioral variations and requires cross-disciplinary, cross-site collaborations for fruitful progress. Teams should include educators and educational psychologists as well as geneticists, neuroscientists, and clinical linguists.

## Glossary of Genetic Terms

**Allele**: a piece of DNA that has two or more possible states in the population.

**Association**: if a certain genetic variant is more common in a group of individuals with a certain trait than in a group of individuals without the trait, it is said to be associated with the trait.

**Categorical affectation status**: term used to label an individual as having a disorder or not.

**Centromeric**: term referring to a location in or near a chromosome’s centromere, a region that serves as a physical connector when two paired chromosomes attach to each other during a part of cell division.

**Chromatin**: the DNA with the proteins that allow it to be condensed in the nucleus.

**Complex disorder**: a disorder thought to result from the effects of multiple genes simultaneously. Environmental factors can influence the disorder as well. This is in contrast to Mendelian disorders, which are caused by disruptions in single genes.

**Copy number variation (CNV)**: regions of the genome that are duplicated or deleted. Some CNVs are polymorphic in the population or do not involve genes and have no discernable phenotype, whereas others cause disease.

**CpG islands**: short DNA sequences that contain a high frequency of neighboring CG dinucleotides. They are usually located at or near the transcription start site of genes. The cytosines in these configurations are subject to methylation. Dense CpG methylation is often associated with silencing of a gene.

**Cytogenetic**: referring to structures and functions of chromosomes in cells.

**Distal**: directional term referring to a location on a chromosome that is closer to chromosome’s end point given a reference point.

**Ectopia**: a collection of cells in the wrong location.

**Endophenotype**: a measurable aspect of a disorder that may not be an obvious symptom of the disorder itself but that has a clear genetic origin.

**Epigenetic**: refers to heritable changes in gene function where the DNA sequence itself remains unchanged.

**Exon**: a portion of a gene that is included in the messenger RNA. Genes are comprised of exons and introns.

**Familial clustering**: the observation of higher prevalence rates of a disorder among biologically related individuals compared with the general population – also referred to as familial aggregation.

**Fluorescence** **in situ hybridization (FISH)**: fluorescent probes that bind specifically to complementary DNA or RNA. Fluorescence microscopy can be used to detect the location of the fluorescent probe on a chromosome or to identify which cell type or tissue expresses the RNA.

**Genome**: the entire DNA sequence in an individual.

**Genome-wide association study (GWAS)**: in samples of individuals with, and without, a trait of interest, markers across the entire genome are tested for association with the trait.

**Haplotype**: a sequence of DNA nucleotides, markers, or genes located in close proximity on a chromosome and inherited together.

**Haploinsufficiency**: when presence of only one working copy of a gene does not produce enough of the protein product to have normal function.

**Heritability**: the proportion of variability of a trait in a sample of individuals that is caused by genetic factors.

**Heterogeneity**: a disorder can look similar across several families, but when different genes cause the disorder in different families, the disorder is heterogeneous.

**Heteromorphism**: an alternate chromosomal structure.

**Heterotopia**: periventricular heterotopia is a condition in which neurons do not migrate properly during fetal brain development and form clumps neat the ventricles rather than moving outward to their intended layer of the cerebral cortex.

**Histone**: proteins that attach to DNA to form chromatin.

**HLA locus**: a region on chromosome 6 where many genes belonging to the human leukocyte antigen (HLA) system reside. This region is of importance for the human immune system.

**Homolog**: genes of similar structure and function are called homologs.

**Intron**: short for “intervening sequence,” the portion of a gene that separates the exons. The introns are removed from the RNA before it is translated into protein.

**Linear association modeling**: testing to determine whether the magnitude of a given trait is correlated with the number of a suspected risk variant. As diploid organisms, humans can have 0, 1, or 2 copies of a risk variant. If individuals with 2 copies show significantly higher levels of the trait than individuals with 1 or 0 copies, there is evidence of genetic association.

**Linkage**: certain genetic variants that are observed in the presence of certain traits are said to be in linkage with each other. Frequently, linkage is due to the fact that genetic regions that are located within close proximity on the same chromosome are inherited together.

**Locus (plural: loci)**: a specific piece of DNA, described by its location on a given chromosome.

**Logarithm of odds (LOD) score**: the likelihood of observing a set of test data given that two genomic regions are linked with each other is compared to the likelihood of obtaining these results in the absence of linkage. The logarithm of this ratio is the log odds score, and it is widely used as a statistic to measure evidence of genetic linkage.

**Long arm**: most chromosomes have a shorter and a longer segment, measured from the endpoint to the centromere. The longer segment or arm is referred to as the q arm, whereas the shorter segment or arm is called the p arm.

**Major allele**: when two or more variants of a piece of DNA exist in the population, the most commonly found variant is called the major allele.

**Methylation**: certain DNA sequences can be modified by addition of a methyl group, comprised of one carbon and three hydrogen atoms, to cytosine – one of the four nucleotide types. When certain parts of a gene are highly methylated, it is inactivated and there is no expression and no protein produced.

**MIM**: acronym for Mendelian Inheritance of Man, a comprehensive catalog of disorders with a genetic etiology. MIM numbers refer to these catalog entries that can be accessed online at OMIM (http://www.ncbi.nlm.nih.gov/omim/).

**Models of transmission**: how a trait is inherited in given families can be estimated by observing the distribution of the trait among the relatives. For instance, if the trait is present in successive generations and approximately half of the children of a person with that trait also have it, an autosomal dominant model may fit this configuration best. An example of this form of inheritance is Huntington disease. Another model of transmission is autosomal recessive, where the trait is usually found in siblings but not in the parents who both carry a risk allele for the trait. An example of this type of inheritance is cystic fibrosis.

**Non-parametric**: non-parametric statistical approaches to evaluating genetic markers for evidence of being inherited along with a disorder do not assume any particular modes of inheritance.

**Parametric**: parametric statistical approaches to evaluating genetic markers for being inherited along with a disorder are based on the parameters of an assumed mode of inheritance, e.g., autosomal dominance.

**Phenotype**: an observable characteristic that may or may not have a genetic origin. A phenotype can be qualitative, such as normal or impaired reading, or quantitative, such as performance on a behavioral test. Both types of phenotypes have been used in genetic studies of reading impairment.

**Pleiotropy**: a single gene can affect multiple observable traits.

**Promoter**: a piece of DNA that is crucial for initiating the process of transcribing a gene’s DNA into RNA.

**Quantitative transmission disequilibrium testing**: testing to determine whether certain genetic variants are transmitted more frequently than expected by chance from an affected parent to an affected child. The term “quantitative” refers to a quantitative characteristic, as opposed to a binary one.

**Recurrence risk**: the risk that a younger sibling of a child with a certain disorder will also have this disorder.

**RNAi, shRNA**: the process of RNA interference (RNAi) uses short RNA molecules that can form a hairpin structure (shRNA). The synthesized shRNAs decrease translation from naturally occurring RNA that contains a complementary sequence.

**Segregate**: a disorder is inherited along with the chromosome on which the causal gene resides. During meiosis to generate sperm and ova, the two copies of a chromosome separate (segregate) into the two haploid gametes that are produced from a single diploid cell.

**Single nucleotide polymorphism (SNP)**: a single base pair that has an alternate form in the population. An individual can carry two copies of one type, two copies of the other type, or one copy of each.

**Splice variants**: after transcription from genomic DNA to mRNA, during which the intronic sequences are removed, one or more protein-coding exons may be spliced out. The different RNA forms are called alternative transcripts and their protein products (isoform) may have different patterns of tissue localization and different activities.

**Structural chromosomal rearrangement**: during meiosis, errors can occur such that pieces of a chromosome are deleted, duplicated, or moved to a different chromosome, all of which result in an altered chromosome structure.

**Targeted genetic studies**: when there are reasons to suspect that a certain gene is causal or that a causal gene resides in a certain part of a chromosome, this gene or genomic region is given preference for genetic analysis over other genes, regions, or even the whole genome.

**Transcription**: the process of producing messenger RNA (mRNA) from its corresponding DNA.

**Transcription factor**: a gene product that regulates the function of another gene.

**Transfect**: insertion of genetic material into cells.

**Translation**: in molecular biology this refers to the process of producing a protein based on the sequence of the corresponding mRNA.

**Untranslated region (UTR)**: pieces of DNA that are part of a gene but that do not code for a protein.

## Conflict of Interest Statement

The authors declare that the research was conducted in the absence of any commercial or financial relationships that could be construed as a potential conflict of interest.
